# Prevalence of metabolic syndrome and the comparison of fasting plasma glucose and HbA1c as the glycemic criterion for MetS definition in non-diabetic population in Ghana

**DOI:** 10.1186/s13098-019-0423-0

**Published:** 2019-03-22

**Authors:** Max Efui Annani-Akollor, Edwin Ferguson Laing, Henry Osei, Evans Mensah, Eddie-Williams Owiredu, Bright Oppong Afranie, Enoch Odame Anto

**Affiliations:** 1 Department of Molecular Medicine, School of Medical Sciences, College of Health Sciences, Kwame Nkrumah University of Science and Technology, Kumasi, Ghana; 2 St Francis Xavier Hospital, Assin Fosu, Central Region Ghana

**Keywords:** Hypertension, Metabolic syndrome, Cardiovascular disease, Fasting plasma glucose, Glycated hemoglobin

## Abstract

**Background:**

Glycated hemoglobin (HbA1c), owing to its ability to reflect glycemia over a relatively longer time span, is still been investigated as an adjunct test for fasting plasma glucose (FPG) to identify subjects at risk of metabolic syndrome (MetS) in some Caucasian populations. However, whether or not HbA1c can serve as an adjunct to FPG in the definition of MetS in the Ghanaian population remains unknown. This study determined the prevalence of MetS and evaluated HbA1c ≥ 5.6% and FPG ≥ 5.6 mmol/l as the glycemic component of MetS among non-diabetic population in Ghana.

**Methods:**

This was a case–control study conducted at St Francis Xavier Hospital, Assin Fosu, Central Region, Ghana. A total of 264 non-diabetic Ghanaian adults consisting of 158 newly diagnosed hypertensives and 106 normotensives, were recruited for the study. Fasting plasma insulin and glucose, HbA1c, and lipid profile was performed for each respondent.

**Results:**

Using the FPG as glycemic criterion, the overall MetS prevalence was 46.6%, 37.1%, and 12.5% according by the IDF, NCEP ATP III, and WHO criteria, respectively. The prevalence of MetS using the HbA1c criterion was 54.2%, 52.7%, and 42.4% by the IDF, NCEP ATP III and WHO criteria, respectively. The HbA1c criterion identified more participants with MetS compared to the FPG criterion with a good agreement between HbA1c and FPG using the IDF and NCEP ATP III criteria (κ = 0.484 to 0.899) respectively. However, the overlap between HbA1c and FPG based diagnosis of MetS was limited for the WHO criterion.

**Conclusion:**

The prevalence of metabolic syndrome is high among non-diabetics in Ghana. Introduction of HbA1c in addition to FPG in the screening of MetS improves identification of more people with MetS who would otherwise have been missed when only FPG-based diagnosis of MetS is used; with a substantial agreement with FPG, except when using the WHO criteria.

**Electronic supplementary material:**

The online version of this article (10.1186/s13098-019-0423-0) contains supplementary material, which is available to authorized users.

## Background

Metabolic syndrome (MetS) is a multiplex risk factor that predisposes persons to the development of diabetes, heart diseases, and stroke [1]. Individuals with MetS have a five-fold and two-fold increase in the risk of type 2 diabetes mellitus (T2DM), and cardiovascular diseases (CVDs), respectively over the next 5–10 years [2]. MetS poses an increasing public health and clinical challenge worldwide in the wake of urbanization, surplus energy intake, increasing obesity and sedentary lifestyle habits [3]. Worldwide prevalence of MetS ranges from < 10% to as high as 84%, depending on the environment factors, sex, age, race, and ethnicity, as well as the method of defining MetS [4, 5].

Studies have shown that MetS contributes to vascular damage in the heart, kidney and brain; similar to adverse effects associated with hypertension [6, 7]. Hypertension is a major global public health problem and contributes to the burden of heart disease, stroke, kidney failure and premature mortality and disability particularly in the developing countries [8–10]. The disease has a global prevalence of 40% and 46% in Africa [11]. In Ghana, the prevalence of hypertension ranges from 19 to 48% [12].

Glycated hemoglobin (HbA1c) reflects the effect of a three (3) month average plasma glucose concentration, based on the length of glycemic episodes and frequency of such glycemic events [13]. Owing to the fact that it can be performed at any time of the day without requiring any special preparation such as an overnight fast, and its ability to reflect glycemia over a relatively longer time span, the American Diabetes Association (ADA) have recommended the use of HbA1c to define the category of increased diabetes risk [14]. Furthermore, according to Park et al. [15], HbA1c may predict incident CVDs even in individuals without diabetes.

Studies conducted in the USA [16], Europe [17, 18], and China [19] have shown that HbA1c may be used in place of fasting plasma glucose (FPG) in identifying individuals with MetS. Nonetheless, with evidence suggesting that HbA1c value differ according to ethnic origin [20–22], it remains unknown whether or not HbA1c can be adjunct to FPG in the definition of MetS in the Ghanaian population. It is against this background that this study estimated the prevalence of MetS and compared HbA1c ≥ 5.6% and FPG ≥ 5.6 mmol/l as the glycemic component of MetS among non-diabetics.

## Materials and methods

### Study design and setting

This was a case–control study conducted at St Francis Xavier Hospital, Assin Fosu, Central Region, Ghana between August, 2015 to November, 2016. The population of Assin North Municipality according to the 2010 Population and Housing Census is 161,341 representing 7.3% of the region’s total population [23].

### Study population

The sample size was calculated using the Raosoft sample size calculator participants at 95% confidence level, 7% margin of error, and a response distribution of 50% [24]. A total of 350 non-diabetic Ghanaian adults, living in Assin Foso were targeted for the study. After excluding participants with diabetes and subjects suspected of acute malignancies, inflammatory disease, and subjects on lipid or glucose-lowering medication, or antihypertensive agents, a total of 264 non-diabetic subjects comprising 158 newly diagnosed hypertensives and 106 normotensives were included in the study.

### Questionnaire administration

Validated questionnaire was used to obtain socio-demographic and lifestyle characteristics of study participants.

### Blood pressure measurement

Blood pressure (BP) was measured with an automated blood pressure apparatus (Omron MX3-Omron Matsusaka Co., Ltd. Japan) by a professional nurse after a research participant has rested for at least 5 min in a sitting position. The average of two readings taken 5 min apart was recorded as the blood pressure measurement. Hypertension was defined as BP ≥ 140/90 mmHg while normotension was defined as BP < 140/90 mmHg [25].

### Anthropometric evaluation

Weight was measured in light clothing without shoes, and in an upright position using a calibrated analogue scale (Seca, Hamburg, Deutschland). Height was measured without shoes using a stadiometer (Seca, Hamburg, Deutschland). Body mass index (BMI) was calculated using the equation; [BMI (kg/m^2^) = weight/height^2^]. Waist circumference (WC) and hip circumference (HC) were measured using a measuring tape. The waist to height ratio (WHtR) = WC (m)/height (m), waist to hip ratio (WHR) = WC (m)/HC (m), body adiposity index (BAI) = (100 × HC (m))/(height (m) × √height (m)) − 18 [26], and visceral adiposity index (VAI) = (WC (m))/(39.68 + (1.88 × BMI)) × [triglyceride (TG)/(1.03)] × ((1.31)/high density lipoprotein cholesterol (HDL-C)) for males and (WC (m))/(36.58 + (1.89 × BMI)) × (TG/(0.81)) × ((1.52)/HDL-C) for females [27] were also calculated. Blood pressure and anthropometric evaluations were carried out on each participant by same trained personnel.

### Sample collection and preparation

From each participant, about five milliliters (5 ml) of venous blood was obtained from the antecubital vein after an 8-h fasting period. One milliliter (1 ml) was dispensed into a fluoride oxalate tube, one milliliter (1 ml) into EDTA tube, and three milliliters (3 ml) into gel separator tubes.

The fluoride oxalate and gel separator tubes were centrifuged at 3000 rpm for 10 min to obtain the plasma and serum. Plasma glucose was measured immediately and the sera for the measurement of other biochemical variables were stored at − 20 °C until analysis (Additional file 1).

### Biochemical assays

Insulin was assayed by sandwich ELISA method (Greenstone Swiss Co. Ltd, China), using a polystyrene microtiter plate [Biobase Biodustry (Shandong) Co., Ltd., China] according to the manufacturer’s instructions. Fasting plasma glucose (FPG), and lipid profile were estimated enzymatically using Mindray BS 120 automated analyzer. Low density lipoprotein cholesterol (LDL-C) concentration was determined using Friedewald’s formula: LDL-C (mmol/L) = total cholesterol (TCHOL) (mmol/L) − high density lipoprotein cholesterol (HDL-C) (mmol/L) − [triglyceride (TG) (mmol/L)/2.2] [28]. Homeostatic model assessment for insulin resistance (HOMA-IR) and beta cell function (HOMA-β) were calculated with calculators provided by the Oxford Centre for Diabetes, Endocrinology and Metabolism [29].

Whole blood was used for HbA1c estimation by turbidimetric inhibition immunoassay using the Cobas Integra automated Chemistry analyzer (Roche Cobas Integra 400 Plus, Roche Diagnostics, USA) [30]. Briefly, well-mixed EDTA-anticoagulated whole blood was put into sample tubes. The tubes were immediately placed on a rack. Red blood cells are then haemolysed by low osmotic pressure and the free haemoglobin subsequently degraded by pepsin to ensure availability of the N-terminal of the beta chain (β-N-terminal) of haemoglobin. Latex particles-bound monoclonal antibodies bind to the β-N-terminal of HbA1c while the remaining free antibodies are agglutinated using synthetic polymers with multiple copies of the β-N-terminal structure of HbA1c. The change in turbidity is measured at 552 nm and the final HbA1c value expressed as a percentage using the formula: HbA1c (%) = (HbA1c/Hb) × 87.6 + 2.27. The test was standardized with an intra-assay %CVs of 0.9–1.5% and inter-assay %CVs of 1.1–1.6%. Daily calibration and maintenance of the analyzer was performed according to the manufacturer’s instructions as previously described [31]. Quality control (QC) was assessed using quality control materials provided by the manufacturer [negative and positive controls (high and low HbA1c)] and calibration was performed using manufacturer-supplied calibrator (Cfas HbA1c).

### Definition of metabolic syndrome

#### National Cholesterol Education Program, Adult Treatment Panel III (NCEP ATP III) criteria

According to the NCEP ATP III, individuals with metabolic syndrome should have at least three of the following: (1) abdominal obesity (WC > 102 cm for male and > 88 cm for female); (2) raised TG (≥ 1.7 mmol/L); (3) low HDL-C (< 1.0 mmol/L in male and < 1.3 mmol/L in female); (4) high blood pressure (BP) (systolic BP ≥ 130 mmHg or diastolic BP ≥ 85 mmHg or treatment of hypertension); and (5) raised fasting plasma glucose (FPG) ≥ 5.6 mmol/L [32, 33].

#### World Health Organization (WHO) criteria

The WHO criteria involve the presence of diabetes mellitus, insulin resistance, or impaired glucose tolerance and any two of the following: (1) BMI ≥ 30 kg/m^2^ and/or WHR > 0.90 for male and > 0.85 for female; (2) high BP (systolic BP ≥ 140 or diastolic BP ≥ 90 mmHg or on medication; (3) TG ≥ 1.7 mmol/L and/or HDL-C < 0.91 mmol/L for male and < 1.01 mmol/L for female [33, 34].

#### International Diabetes Federation (IDF) criteria

According to the IDF criteria, metabolic syndrome is diagnosed if there is central obesity (WC > 94 cm for male and > 80 cm for female) in addition to any two (2) of the following four (4) factors: (1) TG level ≥ 1.7 mmol/L; (2) HDL-C < 1.0 mmol/L for male and < 1.3 mmol/L for female; (3) BP≥ 130/85 mmHg or treatment of previously diagnosed hypertension; and (4) FPG ≥ 5.6 mmol/L or previously diagnosed type 2 diabetes [33, 34].

#### HbA1c criterion for MetS

HbA1c level as glycemic component for MetS was set at ≥ 5.6% [35].

#### Data analysis

The WHO, NCEP ATP III and IDF criteria were used individually to assess the prevalence of MetS. All categorical data were presented as frequencies (percentages) and Chi square and Fisher’s exact test statistic were used to test for association where applicable. Independent t test was used to compare continuous variables between hypertensives and normotensive controls. The kappa (ĸ) statistic was used to evaluate the agreement between FPG- and HbA1c-based identification of MetS. A *p* value < 0.05 was considered statistically significant. All statistical analyses were performed using IBM SPSS 25.0 Statistics.

## Results

Out of the 264 participants recruited for the study, 158 (59.8%) were hypertensives while 106 (40.2%) normotensive. The mean age of the study population was 50.33 years. A higher proportion of the participants were females (59.5%), married (74.6%), had primary education (53.4%). Thirty-three percent (33.0%) were farmers, 95.1% do not smoke, and 86.0% do not take alcoholic beverages (Table 1).

**Table 1:** Crude characteristics of categorical variables of participants

Parameter	Overall	Normotensive; n = 106/40.2%	Hypertensive; n = 158/59.8%	P-value
Age (years)	50.33 ± 10.2	49.77 ± 10.6	50.81 ± 10.3	0.427
Gender				
Females	157 (59.5)	57 (53.8)	100 (63.3)	0.127
Males	107 (40.5)	49 (46.2)	58 (36.7)	
Marital status				
Single	18 (6.8)	14 (13.2)	4 (2.5)	0.007
Married	197 (74.6)	81 (76.4)	116 (73.4)	
Divorced	27 (10.2)	6 (5.7)	21 (13.3)	
Widowed	22 (8.3)	5 (4.7)	17 (10.8)	
Education status				
Illiterate	55 (20.8)	25 (23.6)	30 (19.0)	*0.009*
Primary	141 (53.4)	45 (42.5)	96 (60.8)	
Secondary	54 (20.5)	31 (29.2)	23 (14.6)	
Tertiary	14 (5.3)	5 (4.7)	9 (5.7)	
Major occupation				
Unemployed	19 (7.2)	5 (4.7)	14 (8.9)	*0.023*
Farmer	87 (33.0)	39 (36.7)	48 (30.4)	
Trader	83 (31.4)	23 (21.7)	60 (38.0)	
Nurse	7 (2.7)	4 (3.8)	3 (1.9)	
Teacher	17 (6.4)	7 (6.6)	10 (6.3)	
Beautician	8 (3.0)	6 (5.7)	2 (1.3)	
Other	43 (16.3)	22 (20.8)	21 (13.3)	
Smoking status				
No	251 (95.1)	102 (96.2)	149 (94.3)	0.640
Yes	5 (1.9)	1 (0.9)	4 (2.5)	
Former	8 (3.0)	3 (2.8)	5 (3.2)	
Drinking status				
No	227 (86.0)	77 (72.6)	150 (94.9)	*< 0.0001*
Yes	20 (7.6)	18 (17.0)	2 (1.3)	
Former	17 (6.4)	11 (10.4)	6 (3.8)	

Table 2 displays proportions of MetS components by HbA1c quartiles. Above the first quartile (HbA1c ≤ 5.0%), there were increasing proportions of all MetS components across increasing quartiles of HbA1c. The number of subjects with MetS was in the highest quartile of HbA1c (> 6.0%) in all three criteria (Table 2).

**Table 2:** Proportions of MetS components by HbA1c quartiles for the entire study population

Components	Q1 (≤ 5.0)	Q2 (5.1–5.5)	Q3 (5.6–6.0)	Q4 (> 6.0)
TG (≥ 1.7)	16 (17.0)	16 (17.0)	23 (24.5)	39 (41.5)
HDL-C (male < 1.0, female < 1.3)	25 (19.1)	15 (11.5)	31 (23.7)	60 (45.8)
HDL-C (male < 0.9, female < 1.0)	11 (15.7)	8 (11.4)	15 (21.4)	36 (51.4)
FPG (≥ 5.6)	5 (6.7)	8 (10.7)	25 (33.3)	37 (49.3)
WC (male ≥ 94, female ≥ 80)	30 (16.0)	25 (13.3)	55 (29.3)	78 (41.5)
WC (male ≥ 102, female ≥ 88)	18 (13.3)	20 (14.8)	39 (28.9)	58 (43.0)
WHR (male > 0.90, female > 0.85) and/or BMI ≥ 30	24 (14.5)	21 (12.7)	48 (29.1)	72 (43.6)

*WHR* waist to height ratio, *BP* blood pressure, *BMI* body mass index, *FPG* fasting plasma glucose, *TG* triglycerides, *HDL-C*, high density lipoprotein cholesterol, *WC* waist circumference

The prevalence of MetS components for all participants according to Model 1, and Model 2 is shown in Table 3. Model 2 identified more participants with more MetS components (3 or more components) compared to Model 1 regardless of the MetS diagnostic criteria used (Table 3).

**Table 3:** Prevalence of MetS components according to FPG and HbA1c criteria

Model	No. of components	IDF	NCEP ATP III	WHO
Model 1	0	19 (7.2)	27 (10.2)	36 (13.6)
	1	47 (17.8)	64 (24.2)	48 (18.2)
	2	70 (26.5)	75 (28.4)	81 (30.7)
	3	72 (27.3)	68 (25.8)	62 (23.5)
	4	47 (17.8)	26 (9.8)	32 (12.1)
	5	9 (3.4)	4 (1.5)	5 (1.9)
Model 2	0	3 (1.1)	6 (2.3)	18 (6.8)
	1	41 (15.5)	50 (18.9)	43 (16.3)
	2	65 (24.6)	69 (26.1)	64 (24.2)
	3	79 (29.9)	76 (28.8)	96 (36.4)
	4	60 (22.7)	51 (19.3)	33 (12.5)
	5	16 (6.1)	12 (4.5)	10 (3.8)

Model 1 is the traditional definition of MetS by the various criteria (IDF, NCEP ATP II, and WHO). Model 2 represents a substitution of FPG component in the traditional criteria with HbA1c

Table 4 shows the prevalence of MetS and the assessment of concordance evaluation between FPG and HbA1c in diagnosing MetS. Overall MetS prevalence was 46.6%, 37.1%, and 12.5% according to the traditional IDF, NCEP ATP III, and WHO criteria, respectively using FPG as the glycemic criterion (Model 1). The prevalence of MetS using the HbA1c criterion (Model 2) for the general population were 54.2%, 52.7%, and 42.4%, respectively according to IDF, NCEP ATP III and WHO criteria. Model 2 identified more participants with MetS compared to Model 1. However, varying degrees of overlap between HbA1c and FPG based diagnosis of MetS were observed. A good agreement between HbA1c and FPG criterion was observed for the diagnosis of MetS based on the IDF and NCEP ATP III criteria, with kappa coefficient ranging from 0.484 to 0.899. However, the overlap between HbA1c and FPG based diagnosis of MetS was limited for the WHO criterion. A similar observation was made after stratification by gender (Table 4).

**Table 4:** Prevalence of MetS and concordance evaluation between FPG and HbA1c in diagnosing MetS

MetS criteria	Overall	Female	Male
IDF			
Model 1	123 (46.6)	92 (58.6)	31 (29.0)
Model 2	143 (54.2)	107 (68.2)	36 (33.6)
Overlap	117 (44.3)	87 (55.4)	30 (28.0)
Kappa	0.759	0.660	0.848
NCEP ATP III			
Model 1	98 (37.1)	74 (47.1)	24 (22.4)
Model 2	139 (52.7)	100 (63.7)	39 (36.4)
Overlap	98 (37.1)	74 (47.1)	24 (22.4)
Kappa	0.694	0.674	0.670
WHO			
Model 1	33 (12.5)	24 (15.3)	9 (8.4)
Model 2	112 (42.4)	63 (40.1)	49 (45.8)
Overlap	20 (7.6)	14 (8.9)	6 (5.6)
Kappa	0.103	0.129	0.075

Model 1; traditional FPG criteria. Model 2; traditional criteria with the glycemic component replaced by HbA1c. The kappa (ĸ) statistic was used to evaluate the agreement between FPG-(model 1) and HbA1c-based identification of MetS (model 2), Overlap; Diagnosing of same patient with MetS by both FPG and HbA1c

## Discussion

Glycated haemoglobin (HbA1c) is formed by irreversible glycation of the N-terminal valines of the β-chains of haemoglobin [36] and reflects the average glycemia over a period of 3 months [37]. Its measurement is not limited by the requirement of a fasting sample, which confers some advantages over fasting plasma glucose.

As a result, the American Diabetes Association recommends that screening for pre-diabetes should include HbA1c. In their a study, they found that individuals with HbA1c within 5.7–6.4% are at high risk for diabetes [38]. Additionally, Blake et al. [39] in a case–control study reported that high baseline HbA1c could predict future cardiovascular events in non-diabetic women by a factor of 2.25. Osei et al. also reported that, when HbA1c of non-diabetic, first-degree relatives of African-American type 2 diabetes mellitus (T2DM) patients were stratified by tertiles of 4.7% (3.3–4.8%), 5.4% (4.9–5.6%), and 5.8% (5.7–6.4%), individuals with HbA1c level within 5.7–6.4% had increased number of some metabolic syndrome (MetS) components, which buttresses the influence of HbA1c in MetS diagnosis. In another study among non-diabetic Korean adults, Sung and Rhee reported that insulin resistance, which is the mechanism underpinning the aetiology of metabolic syndrome, increased with increasing quartile of HbA1c [40]. In the present study, we compared MetS using HbA1c or fasting plasma glucose (FPG) as the glycemic component among non-diabetic patients. To the best of our knowledge, this is the first study comparing the HbA1c and FPG as the glycemic component of MetS among non-diabetic population in Ghana.

In order to assess the relationship between HbA1c and MetS components, we evaluated the proportions of the MetS components by HbA1c quartiles. We observed that above the first quartile (HbA1c ≤ 5.0%), there were increasing proportion of MetS components across increasing HbA1c quartiles, with the most number of MetS subjects being in the highest quartile of HbA1c (> 6.0%) in all three criteria used. This suggests that elevated HbA1c may predispose to dysmetabolism as consistent with previous studies [39, 41]. In this study, we also identified more individuals with higher number of MetS components (3 or more components) using HbA1c-based criteria compared to the traditional FPG-based criterion; the prevalence of MetS using the HbA1c criteria was higher compared to the FPG criteria across all three criteria. This finding may be due to the robust association between HbA1c and the components of MetS compared to FPG. Saravia et al. in a cross-sectional study among 3200 non-diabetic male participants in the Aragon Workers’ Health Study observed that HbA1c was closely associated with high waist circumference, elevated triglycerides, and reduced HDL-C compared to FPG [42]. Succurro et al. in a study cohort among Italian nondiabetic white subjects also reported that HbA1c better correlated with visceral obesity, HDL-C, and triglycerides than FPG [17]. These findings suggest that introduction of the HbA1c criterion during screening for MetS may contribute to identification of more individuals at risk of cardiovascular events among non-diabetics who would have otherwise be considered normal [43]. Nevertheless, it is noteworthy that varying degrees of overlap between HbA1c and FPG-based diagnosis of MetS was observed. There was a good agreement between HbA1c and FPG criteria for the diagnosis of MetS based on the IDF and NCEP ATP III criteria and this corroborates the finding from a case–control study by Siu and Yuen among Hong Kong Chinese adults [44] who reported that applying the HbA1c criterion improved the identification of subjects with MetS by 13% compared with FPG; with HbA1c having a good agreement with the FPG criterion (90.7%, κ = 0.62). A study by Ong et al. among United States adults also reported an increased level of agreement of 91.3% between HbA1c and FPG in diagnosing MetS [16]. A similarly increased identification of MetS subjects using HbA1c, in addition to a good agreement with FPG was observed by Janghorbani et al. in an Iranian population [45] and Bernal-Lopez et al. among a Mediterranean urban population from Southern Europe [18]. However, the overlap between HbA1c and FPG-based diagnosis of MetS in this study was limited using the WHO criterion; a finding which is in dissonance with a cross-sectional study by Sun et al. [19] among Chinese participants. This discrepancy may however be attributed to the influence of ethnicity, socio-demographic, and lifestyle disparities on HbA1c values.

Nonetheless, as with most epidemiological studies, the use of point measurement of biochemical parameters is a limitation of this study. Additionally, the study was conducted in a single urban setting and might not be representative of the whole country though we used a large tertiary hospital which serves individuals from different areas. Thus, a larger scale study to confirm this association is warranted.

## Conclusion

The prevalence of metabolic syndrome is high among non-diabetics in Ghana. Introduction of HbA1c in addition to FPG in the Screening of MetS improves identification of more people with MetS who would otherwise have been missed when only FPG-based diagnosis of MetS is used; with a substantial agreement with FPG, except when using the WHO criteria.

## Additional file

**Additional file 1.** Anthropometric, hemodynamic, and biochemical profile of the entire study population; stratified by HbA1c quartiles; and the proportions of MetS components by HbA1c Quartiles stratified by blood pressure status.

## Abbreviations

MetS: metabolic syndrome
ELISA: enzyme-linked immunosorbent assay
T2DM: type 2 diabetes mellitus
LDL-C: low density lipoprotein cholesterol
CVD: cardiovascular disease
HDL-C: high density lipoprotein cholesterol
HbA1c: glycated haemoglobin
TG: triglyceride
FPG: fasting plasma glucose
VLDL-C: very low density lipoprotein cholesterol
BMI: body mass index
HOMA-IR: homeostatic model assessment for insulin resistance
WC: waist circumference
HOMA-B: homeostatic model assessment for beta cell function
HCL: hip circumference
SBP: systolic blood pressure
WHR: waist-to-hip ratio
DBP: diastolic blood pressure
WHtR: waist-to-height ratio
NCEP-ATP III: National Cholesterol Education Program, Adult Treatment Panel III
BAI: body adiposity index
WHO: World Health Organization
VAI: visceral adiposity index
IDF: International Diabetes Federation
