# Supplementary material for: Prevalence of metabolic syndrome and the comparison of fasting plasma glucose and HbA1c as the glycemic criterion for MetS definition in non-diabetic population in Ghana
Source: Diabetol Metab Syndr. 2019 Mar 22;11:26. doi: 10.1186/s13098-019-0423-0 (PMC6431006; doi:10.1186/s13098-019-0423-0)
Supplement: Supplementary file 1 — Additional file 1. Anthropometric, hemodynamic, and biochemical profile of the entire study population; stratified by HbA1c quartiles; and the proportions of MetS components by HbA1c Quartiles stratified by blood pressure status. [file 13098_2019_423_MOESM1_ESM.docx]

**Prevalence of Metabolic syndrome and the comparison of fasting plasma glucose and HbA1c as the glycemic criterion for MetS definition in non-diabetic population in Ghana**

**ADDITIONAL DATA**

The anthropometric, hemodynamic, and biochemical profile of the study population is shown in **Table S1**. Except for VAI, and HOMA-β, there were statistically significant elevations in all anthropometric, hemodynamic and biochemical parameters among hypertensives compared with normotensives. Upon gender stratification, similar presentation was observed in both sexes, with the exception of VAI, TG, HDL-C, very high density lipoprotein cholesterol (VLDL-C), and HOMA-β which were not statistically significant (**Table S1**).

**Table S1. Anthropometric, hemodynamic, and biochemical profile of the study population**

| **Parameter** |  | **Overall** | | **Female** | | **Male** | |
| --- | --- | --- | --- | --- | --- | --- | --- |
|  | **Overall** | **Normotensive** | **Hypertensive** | **Normotensive** | **Hypertensive** | **Normotensive** | **Hypertensive** |
| BMI (kg/m^2^) | 26.49 ± 5.2 | 25.19 ± 4.6 | 27.39 ± 5.4*** | 26.66 ± 4.9 | 28.74 ± 6.0^^ | 23.64 ± 3.7 | 25.48 ± 3.5†† |
| WHR | 0.91 ± 0.1 | 0.89 ± 0.1 | 0.92 ± 0.1** | 0.89 ± 0.1 | 0.91 ± 0.1^ | 0.90 ± 0.1 | 0.92 ± 0.1† |
| WHtR | 0.58 ± 0.1 | 0.56 ± 0.1 | 0.6 ± 0.1*** | 0.59 ± 0.1 | 0.62 ± 0.1^^ | 0.52 ± 0.1 | 0.56 ± 0.1†† |
| BAI | 31.89 ± 6.8 | 30.27 ± 6.4 | 33.03 ± 6.9*** | 34.61 ± 5.3 | 36.35 ± 6.6^ | 26.04 ± 4.1 | 28.60 ± 4.0† |
| VAI | 1.86 ± 2.7 | 1.8 ± 2.1 | 1.9 ± 3.0 | 2.22 ± 2.2 | 2.24 ± 1.8 | 1.42 ± 1.8 | 1.48 ± 4.3 |
| SBP (mmHg) | 133.14 ± 22.3 | 118.47 ± 13.4 | 143.92 ± 20.9*** | 119.12 ± 13.8 | 141.78 ± 18.7^^^ | 117.75 ± 13.0 | 147.18 ± 23.6††† |
| DBP (mmHg) | 82.44 ± 12.37 | 75.76 ± 9.6 | 87.21 ± 11.8*** | 75.69 ± 9.7 | 87.29 ± 10.8^^^ | 75.84 ± 9.6 | 87.10 ± 13.3††† |
| TCHOL (mmol/L) | 4.50 ± 1.1 | 4.24 ± 1.1 | 4.68 ± 1.0*** | 4.36 ± 1.1 | 4.82 ± 1.0^^ | 4.12 ± 1.1 | 4.47 ± 1.0†† |
| TG (mmol/L) | 1.29 ± 0.7 | 1.2 ± 0.7 | 1.35 ± 0.7* | 1.23 ± 0.7 | 1.37 ± 0.6 | 1.18 ± 0.6 | 1.32 ± 0.7 |
| HDL-C (mmol/L) | 1.17 ± 0.4 | 1.14 ± 0.4 | 1.19 ± 0.4 | 1.15 ± 0.4 | 1.16 ± 0.4 | 1.13 ± 0.4 | 1.24 ± 0.4 |
| LDL-C (mmol/L) | 2.93 ± 1.0 | 2.7 ± 1.0 | 3.08 ± 1.0** | 2.81 ± 1.1 | 3.26 ± 1.0^^ | 2.59 ± 0.9 | 2.84 ± 1.1†† |
| VLDL-C (mmol/L) | 0.58 ± 0.3 | 0.55 ± 0.3 | 0.61 ± 0.3* | 0.57 ± 0.3 | 0.63 ± 0.3 | 0.53 ± 0.3 | 0.59 ± 0.3 |
| FPG (mmol/L) | 5.11 ± 0.7 | 4.87 ± 0.8 | 5.28 ± 0.7*** | 4.97 ± 0.7 | 5.28 ± 0.7^^ | 4.77 ± 0.8 | 5.27 ± 0.7†† |
| Insulin (mU/L) | 15.12 ±8.9 | 12.9 ± 8.1 | 17.02 ± 9.1*** | 12.96 ± 9.4 | 17.31 ± 9.4^^ | 12.83 ± 8.1 | 16.60 ± 8.6†† |
| HOMA-IR | 3.43 ± 2.3 | 2.77 ± 1.9 | 4.01 ± 2.4*** | 2.81 ± 1.9 | 4.18 ± 2.5^^^ | 2.74 ± 2.0 | 3.76 ± 2.1††† |
| HOMA-β | 213.86 ±196.9 | 226.2 ± 241.9 | 205.23 ± 151.8 | 194.27 ± 224.3 | 209.65 ±164.3 | 260.71 ± 252.6 | 199.07 ± 132.3 |
| HbA1c (%) | 5.76 ± 1.1 | 5.32 ± 0.9 | 6.09 ± 1.2*** | 5.35 ± 0.8 | 6.23 ± 1.3^^^ | 5.29 ± 0.9 | 5.88 ± 1.0††† |

*Significant at the 0.05 level, **significant at the 0.01 level and ***significant at the 0.001 level when normotensives are compared with the hypertensives. **^**significant at the 0.05 level, **^^**significant at the 0.01 level and **^^^**significant at the 0.001 level when female normotensives are compared with female hypertensives. **†**significant at the 0.05 level, **††**significant at the 0.01 level and **†††**significant at the 0.001 level when male normotensives are compared with male hypertensives. SBP, Systolic blood pressure. DBP, Diastolic blood pressure. BMI, Body Mass Index. BAI, Body Adiposity Index. VAI, Visceral Adiposity Index. FPG, Fasting plasma glucose. TCHOL, Total Cholesterol. TG, Triglycerides. HDL-C, High Density Lipoprotein cholesterol. LDL-C, Low Density Lipoprotein cholesterol. VLDL-C, Very High Density Lipoprotein cholesterol. HOMA-IR, Homeostatic Model Assessment for Insulin Resistance. HOMA- β, Homeostatic Model Assessment for beta cell function

**Table S2** shows the Anthropometric, hemodynamic, and biochemical profiles of the study population stratified by HbA1c quartiles. There was statistically significant elevated systolic blood pressure (SBP), and diastolic blood pressure (DBP) among hypertensives compared with normotensives across increasing quartiles of HbA1c. There was a statistically significant higher WHtR, BAI, and TCHOL among hypertensives compared with normotensives with respect to HbA1c of 5.6-6.0%, and TCHOL among subjects with HbA1c >6.0%. BMI, BAI, Insulin, and HOMA-IR increased across increasing HbA1c quartiles among the normotensives though not statistically significant (**Table S2**).

**Table S2. Anthropometric, hemodynamic, and biochemical profile of the study population stratified by HbA1c quartiles**

| Parameter | Q1 ( ≤ 5.0 ) | | Q2 (5.1-5.5) | | Q3 (5.6-6.0) | | Q4 ( > 6.0) | |
| --- | --- | --- | --- | --- | --- | --- | --- | --- |
|  | Normotensive | Hypertensive | Normotensive | Hypertensive | Normotensive | Hypertensive | Normotensive | Hypertensive |
| BMI (kg/m^2^) | 24.30 ± 4.7 | 24.66 ± 4.4 | 25.56 ± 2.8 | 26.49 ± 4.4 | 26.26 ± 4.9 | 28.37 ± 6.7 | 27.04 ± 5.1 | 28.14 ± 5.1 |
| WHR | 0.88 ± 0.1 | 0.92 ± 0.1* | 0.92 ± 0.1 | 0.94 ± 0.1 | 0.91 ± 0.1 | 0.92 ± 0.1 | 0.89 ± 0.1 | 0.91 ± 0.1 |
| WHtR | 0.54 ± 0.1 | 0.55 ± 0.1 | 0.57 ± 0.1 | 0.58 ± 0.1 | 0.57 ± 0.1 | 0.61 ± 0.1* | 0.57 ± 0.1 | 0.60 ± 0.1 |
| BAI | 29.11 ± 5.8 | 29.06 ± 5.2 | 29.82 ± 6.1 | 30.02 ± 8.3 | 31.45 ± 5.1 | 34.61 ± 6.4* | 31.78 ± 8.0 | 33.28 ± 6.5 |
| VAI | 1.45 ± 1.8 | 1.28 ± 2.1 | 2.22 ± 1.8 | 1.70 ± 1.3 | 1.80 ± 1.6 | 2.10 ± 5.4 | 2.47 ± 2.7 | 1.93 ± 1.7 |
| SBP (mmHg) | 112.81 ± 14.4 | 136.57 ± 17.8*** | 125.70 ± 11.6 | 144.18 ± 21.8** | 118.18 ± 12.1 | 145.05 ± 24.0*** | 121.67 ± 10.6 | 145.53 ± 18.9*** |
| DBP (mmHg) | 72.81 ± 9.2 | 85.01 ± 11.7*** | 80.00 ± 9.2 | 86.53 ± 8.9** | 72.95 ± 9.8 | 86.86 ± 12.2*** | 77.23 ± 8.0 | 88.65 ± 11.4*** |
| TCHOL (mmol/L) | 3.89 ± 1.1 | 4.31 ± 1.1 | 4.59 ± 1.1 | 5.03 ± 1.0 | 4.46 ± 1.2 | 4.79 ± 1.2 | 4.10 ± 1.0 | 4.63 ± 0.9* |
| TG (mmol/L) | 1.05 ± 0.6 | 1.10 ± 0.5 | 1.45 ± 0.7 | 1.34 ± 0.5 | 1.23 ± 0.7 | 1.43 ± 0.6 | 1.35 ± 0.7 | 1.38 ± 0.7 |
| HDL-C (mmol/L) | 1.16 ± 0.4 | 1.33 ± 0.5 | 1.11 ± 0.3 | 1.23 ± 0.4 | 1.16 ± 0.4 | 1.24 ± 0.4 | 0.98 ± 0.3 | 1.15 ± 0.4 |
| LDL-C (mmol/L) | 2.37 ± 1.1 | 2.62 ± 1.1 | 3.02 ± 1.1 | 3.50 ± 0.9 | 2.92 ± 0.9 | 3.25 ± 0.9 | 2.65 ± 1.0 | 3.08 ± 1.0 |
| FPG (mmol/L) | 4.54 ± 0.6 | 4.82 ± 0.4 | 4.67 ± 0.7 | 5.02 ± 0.6 | 5.27 ± 0.6 | 5.38 ± 0.5 | 5.19 ± 0.7 | 5.33 ± 0.7 |
| Insulin (mU/L) | 10.32 ± 7.4 | 10.60 ± 3.7 | 14.06 ± 8.2 | 15.03 ± 7.5 | 14.08 ± 7.1 | 17.50 ± 8.1 | 18.02 ± 8.3 | 19.35 ± 9.6 |
| HOMA-IR | 2.08 ± 1.6 | 2.69 ± 1.9 | 2.93 ± 1.8 | 3.28 ± 1.6 | 3.30 ± 1.7 | 3.86 ± 2.1 | 4.16 ± 2.1 | 4.84 ± 2.5 |

*Significant at the 0.05 level, **significant at the 0.01 level and ***significant at the 0.001 level when normotensives are compared with the hypertensives. SBP, Systolic blood pressure. DBP, Diastolic blood pressure. BMI, Body Mass Index. BAI, Body Adiposity Index. VAI, Visceral Adiposity Index. FPG, Fasting plasma glucose. TCHOL, Total Cholesterol. TG, Triglycerides. HDL-C, High Density Lipoprotein cholesterol. LDL-C, Low Density Lipoprotein cholesterol. HOMA-IR, Homeostatic Model Assessment for Insulin Resistance.

**Table S3** displays proportions of MetS components by HbA1c quartiles stratified by blood pressure status. A trend where normotensives had a higher proportion of MetS components compared with hypertensives at lower HbA1c values (≤ 5.0% and 5.1-5.5%) and an inverse association where hypertensives had a higher proportion of MetS components compared with normotensives at higher HbA1c levels (5.6-6.0% and >6.0%) was observed, except for the WHO obesity criterion [WHR (Male >0.90, Female>0.85) and/or BMI ≥30 kg/m^2^], where there were increased proportion of MetS components among hypertensives compared with normotensives across the increasing HbA1c quartiles (**Table S3**).

**Table S3. Proportions of MetS components by HbA1c Quartiles stratified by blood pressure status**

| Parameter | Q1 ( ≤ 5.0 ) | | Q2 (5.1-5.5) | | Q3 (5.6-6.0) | | Q4 ( > 6.0) | |
| --- | --- | --- | --- | --- | --- | --- | --- | --- |
|  | Normotensive | Hypertensive | Normotensive | Hypertensive | Normotensive | Hypertensive | Normotensive | Hypertensive |
| TG(≥1.7) | 11 (25.6) | 4 (21.1) | 10 (50.0) | 6 (40.0) | 8 (38.1) | 15 (31.9) | 11 (45.8) | 28 (37.3) |
| HDL(Male<1.03, Female<1.292) | 20 (46.5) | 5 (26.3) | 10 (50.0) | 5 (33.3) | 10 (47.6) | 21 (44.7) | 16 (66.7) | 44 (58.7) |
| HDL(Male<0.9, Female<1.0) | 9 (20.9) | 2 (10.5) | 6 (30.0) | 2 (13.3) | 6 (28.6) | 9 (19.1) | 12 (50.0) | 24 (32.0) |
| FPG(≥ 5.6) | 5 (11.6) | 0 (0.0) | 4 (20.0) | 4 (26.7) | 9 (42.9) | 16 (34.0) | 9 (37.5) | 28 (37.3) |
| WC(Male ≥ 94, Female ≥80) | 23 (53.5) | 7 (36.8) | 15 (75.0) | 10 (66.7) | 15 (71.4) | 40 (85.1) | 16 (66.7) | 62 (82.7) |
| WC(Male ≥102, Female ≥88) | 13 (30.2) | 5 (26.3) | 12 (60.0) | 8 (53.3) | 9 (42.9) | 30 (63.8) | 11 (45.8) | 47 (62.7) |
| WHR(Male >0.90, Female>0.85) and/or BMI ≥30 | 12 (27.9) | 12 (63.2)** | 8 (40.0) | 13 (86.7)** | 8 (38.1) | 40 (85.1)*** | 11 (45.8) | 61 (81.3)** |

*Significant at the 0.05 level, **significant at the 0.01 level and ***significant at the 0.001 level when normotensives are compared with the hypertensives. WHR, Waist to height ratio. BP, blood pressure. BMI, Body Mass Index. FPG, Fasting plasma glucose. TG, Triglycerides. HDL-C, High Density Lipoprotein cholesterol. WC, Waist circumference.
